# Behavior problems associated with brain heterotopia

**DOI:** 10.1192/j.eurpsy.2021.1696

**Published:** 2021-08-13

**Authors:** M. Budisteanu, S. Papuc, A. Erbescu, C. Iliescu, M. Dobre, D. Barca, O. Tarta-Arsene, C. Motoescu, A. Dica, C. Sandu, C. Anghelescu, D. Craiu, A. Arghir

**Affiliations:** 1 Psychaitry Research Laboratory, Obregia Clinical Hospital of Psychiatry, Bucharest, Romania; 2 Genetics Laboratory, ‘Victor Babes’ National Institute of Pathology, Bucharest, Romania

**Keywords:** hyperkinesia with attention deficit, autism, Epilepsy, brain heterotopia

## Abstract

**Introduction:**

Brain heterotopia represent a group of rare malformations with a heterogeneous phenotype, ranging from asymptomatic to severe clinical picture (resistant epilepsy, severe developmental delay). The etiology is multifactorial, including both genetic and environmental factors.

**Objectives:**

In this paper we present our experience regarding behavior problems in patients with heterotopia.

**Methods:**

A cohort of 16 pediatric patients with brain heterotopia, six females and ten males, with age at last follow-up ranging from 2 months to 24 years were investigated by clinical examination, electroencephalographic studies, brain imaging, and genomic tests. Specific psychological tests and psychiatric evaluation were performed in all children for behavior problems assessment.

**Results:**

Six individuals presented behavioral problems: autism (three patients) and hyperkinesia with attention deficit (three patients). All of them had intellectual disability or learning problems; five patients had epilepsy, with drug-resistant seizures in four cases. In two cases the behavioral problems occurred before the onset of epileptic seizures.

**Conclusions:**

Behavior problems are important features in patients with brain heterotopia, making the management of these patients more difficult, especially when they occur in association with drug-resistant epilepsy. Acknowledgements: This work was supported partially by grants of the Romanian National Authority for Scientific Research and Innovation CCCDI – UEFISCDI, Projects COFUND-ERANET E-RARE 3-HETER-OMICS-2 Number 87/2019 and 88/2019 within PNCDI III.

**Disclosure:**

No significant relationships.

